# Decontamination of Dental Implant Surfaces by the Er:YAG Laser Beam: A Comparative in Vitro Study of Various Protocols

**DOI:** 10.3390/dj6040066

**Published:** 2018-12-01

**Authors:** Rima Nejem Wakim, Melanie Namour, Hoang Viet Nguyen, Andre Peremans, Toni Zeinoun, Alain Vanheusden, Eric Rompen, Samir Nammour

**Affiliations:** 1 Department of Oral and Maxillofacial Surgery, School of Dentistry, Lebanese University, Beirut 27798, Lebanon; rimawbta@hotmail.com (R.N.W.); zeinountoni@gmail.com (T.Z.); 2 Department of Dental Science, Faculty of Medicine, University of Liege, 4020 Liege, Belgium; melanienamour@gmail.com (M.N.); nguyen.vietdc@gmail.com (H.V.N.); alain.vanheusden@chu.ulg.ac.be (A.V.); eric.rompen@chu.ulg.ac.be (E.R.); 3 Centre de Recherche en Physique de la Matière et des Rayonnements, Facultés Universitaires Notre Dame de la Paix, 5000 Namur, Belgium; andre.peremans@gmail.com

**Keywords:** carbon, cleaning, Er:YAG laser, implant, irradiation, multiple passages, peri-implantitis

## Abstract

Oral rehabilitation with dental implants has revolutionized the field of dentistry and has been proven to be an effective procedure. However, the incidence of peri-implantitis has become an emerging concern. The efficacy of the decontamination of the implant surface, by means of lasers, is still controversial. Previous studies have revealed a reduction in osteoblast adhesion to carbon-contaminated implant surfaces. This in-vitro study aimed to evaluate the decontamination of failed implants by assessing the carbon proportion, after irradiation by low-energy erbium yttrium-aluminum-garnet laser (Er:YAG) (Fotona; 2940 nm, Ljubljana, Slovenia) for a single and for multiple passages, until getting a surface, free of organic matters; to find the appropriate procedure for dental-implant surface-decontamination. Ninety implants were used. Thirty sterile implants were kept as a negative control. Thirty failed implants were irradiated by the Er:YAG laser, for a single passage, and the other thirty, for multiple passages. The parameters used in our experiments were an irradiation energy of 50 mJ, frequency of 30 Hz, and an energy density of 3.76 J/cm^2^. A sapphire tip, with a length of 8 mm, was used with concomitant water spray irrigation, under air 6 and water spray 4. Super short pulse mode (SSP) was of 50 μs; irradiation speed being 2 mm/s. We used energy-dispersive X-ray spectroscopy (EDX) to evaluate the carbon proportion on the surfaces of the sterile implants, the contaminated, and the lased implants, with one (LX1) and with three passages (LX3). Statistical analysis was performed by ANOVA. Results showed mean difference between the three groups (contaminated, LX1, and LX3) with *p* < 0.0001, as between LX1 and Group A (*p* < 0.0001), while the difference between LX3 and the control group was not statistically significant. The decontamination of the implant surfaces with a low-energy Er:YAG laser with three passages, appeared to be an encouraging approach.

## 1. Introduction

Peri-implantitis is an inflammatory process that takes place in the soft tissue, with a bone loss around an osseointegrated implant in function [1,2]. Diagnosis is based on the bleeding and the probing depth of the peri-implant pockets, along with the suppuration and gradual loss of bone height around the implant [3,4,5]. The etiology of the implant infection includes many factors, such as implant design, degree of roughness, and the status of the tissue surrounding the implant [5]. However, bacterial infection plays the most important role in the failure of dental implants [6]. Many acidogenic bacteria collaborate to produce acidic conditions [7,8] which could promote bacterial survival from organic matters present at the implant surface [9]. Lausma et al. [10] concluded in their study that the presence of large variations in carbon loads (20–60%) on the contaminated implant surface. Shibli et al. [11] showed that there are varying degrees of foreign carbon on the surface of the failed titanium dental implants. Subsequently, bone-to-titanium contact is affected by the accumulation of organic molecules on the implant surfaces [12,13]. As carbon is the most prominent contaminant on titanium surfaces, Hayashi [14] in his study, focused on the effect of carbon adsorption on the bioactivity of titanium; he concluded that the elimination of hydrocarbons seems to be an important step in promoting the bioactivity and osseointegration of titanium. Thereafter, to increase the surface energy and wettability, implant surface must be treated to reduce the degree of hydrocarbon [15]. A general rule has been set that, cleaner is better [16].

Many treatments have been proposed in the literature [17,18,19,20,21,22,23,24,25,26], but until now no methodology has been proven to be a gold standard approach. The decontamination of such surfaces can be problematic, especially as the complex modern surface topography of implants offers an outstanding haven for bacterial adhesion and colonization. The main objective of the treatment is to eliminate soft and hard deposits, without changing the topography, because a surface damage induces changes in the oxide layer and this may impair the osseointegration of the implant [27,28,29,30,31]. Therapeutic strategies comprise surgical and nonsurgical protocols. They include debridement by different means—air-powder abrasive technique, chlorhexidine, tetracycline, metronidazole, citric acid application, and photodynamic therapy [5]. Finally, laser which is a non-invasive method could be used to reduce microorganisms in the peri-implantitis [6,7,8,9,10,11,12,13,14,15,16,17,18,19,20,21,22,23,24,25,26,27,28,29,30,31,32]. Data related to the effects of laser on the biological and surface properties of titanium, are conflicting [33,34,35,36,37]. Many studies have shown alteration in the implant surface resulting from a laser treatment [38], which may be a result of beam absorption by the titanium, with different settings used during studies [37,39,40].

Erbium yttrium-aluminum-garnet laser (Er:YAG) lasers with a wavelength of 2940 nm has been often evaluated as a treatment option for the removal of biofilms from contaminated dental implants [41]. Its laser light is well-absorbed by biofilms and it does not affect the implant surface at a low-energy level [15]. The question still exists regarding what would be an efficient protocol to use, without damaging the implant surface.

Most studies estimated the efficacy of laser decontamination, either by assessing the removal of subgingival calculus or cytotoxic bacterial components from the implant surfaces. There are yet no publications that have evaluated the efficiency of implant cleaning through the detection of carbon atoms. Carbon is the witness to the persistence of any non-biocompatible matter (organic and calcic) that may alter the new osseointegration of the decontaminated implant surfaces.

Since the presence of contamination on the implant surfaces affects the osteoblast adhesion, in this study we aim to assess the efficiency of the Er:YAG laser in removing carbon traces (witnesses to the contamination), through an energy-dispersive X-ray spectroscopy (EDX). The SEM is used to check the elimination of the contaminants and to detect any implant surface alteration. For this purpose, we used a low-energy level of the Er:YAG laser (50 mJ), since at this level there are no morphological changes of the irradiated implant and the elevation of the surface temperature is negligible, with the use of water-cooling [38]. To evaluate the efficiency of the cleaning, we used a single and multiple passages of laser irradiation. The null hypothesis was that the cleanliness of the implants irradiated by the Er:YAG laser was comparable to that of the control group of sterile implants. Thus, there was no significant difference between the irradiated and the control group concerning the count of carbon.

## 2. Materials and Methods

Ninety implants, with a surface that was treated by sandblasting, with titanium oxide, and etched with nitric and hydrofluoric acids (Titanium grade IV-EuroTechnica group, Sallanches, France), were used in our study. They were divided into two groups: Group A (the control group) and Group B (the test group). Group B was then subdivided into two subgroups, based on the decontamination method. This distribution is shown in Table 1.

- Group A: Thirty sterile implants served as the control group. The implants of this group were kept in their own sterile packages, until their examination through EDX and SEM.
- Group B: Sixty contaminated implants were collected from failed cases of implants. The implants were retrieved from patients previously diagnosed with peri-implantitis. Their removal was not, in any of the cases, related to our study (severe peri-implantitis and bone resorbtion, loss of osteointegration, etc.). Before experimentation and for the purpose of standardization, all contaminated implants were preserved in sterile saline liquid of 0.9% NaCl, at a temperature of 37 °C, for the simulation of the intra-oral in vivo conditions; the solution was changed every 24 h, until experimentation. To assess the efficacy of the laser irradiation, in our study we compared the carbon percentage of the contaminated implant surfaces, before and after the laser irradiation. At the baseline, the sixty implants of Group B were all evaluated (eight points were randomly analyzed per sample), using energy dispersive X-ray analysis (SEM–EDX). After that, the implants were randomly assigned into two equal samples—LX1 and LX3—for the laser irradiation. Sample LX1 was irradiated by one passage and sample LX3, by three passages. Afterward, a second analysis of the carbon content of both samples was done.

### 2.1. Decontamination

**Figure 1:**
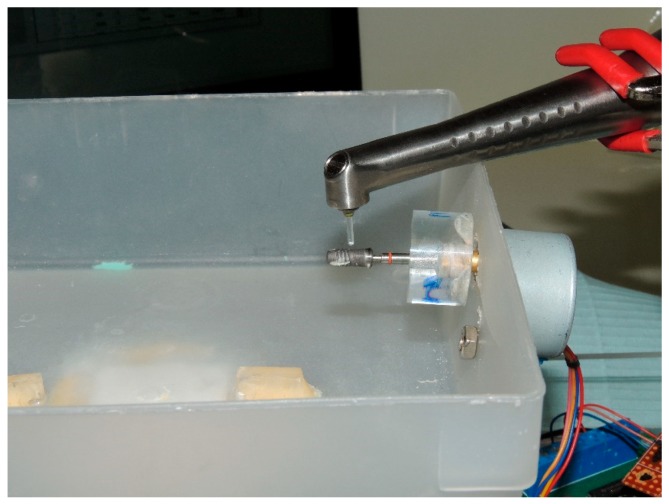
The custom-designed device.

Laser surface preparation: The contaminated implants of Group B were irradiated using a custom-designed device (Figure 1). The custom made machine was driven by a stepper motor, controlled by a software, and was connected to a computer through a USB. Such a device was used for the fixation of the laser handpiece, in a standard manner, for the irradiation of the implant, at a constant speed and time. The machine allowed for the standardization of all laser irradiations, thus, allowing an accurate comparison between the different treatment protocols. It should have been able to:

(1) Standardize the angulation of the laser beam.
(2) Standardize the distance between the tip of the handpiece and the implant surface.
(3) Standardize the exposure time.
(4) Have a semi-adjustable base on which an implant, connected to an abutment, is attached to a Plexiglass^®^ plate.

An Er:YAG laser (Fotona; 2940 nm, Ljubljana, Slovenia) was used in our experiments, with an irradiation energy of 50 mJ, frequency of 30 Hz, output power of 1.5 W, and an energy density of 3.76 J/cm^2^. The energy was chosen, based on the literature review; higher energy was shown to damage the titanium implant surface [38]. A sapphire tip with a length of 8 mm was used in the respective handpiece (H14) and not in contact with the surface of the titanium, with concomitant water spray irrigation, under air 6 and water spray 4, the irradiation angle was 90 degrees, at a focal distance of 2 mm, the spot size diameter was 1.3 mm; Super short pulse mode (SSP) was of 50 μs; the contaminated implants of Group B were irradiated as:

- Thirty contaminated implants received one passage of laser (LX1).
- Thirty contaminated implants received three passages of laser (LX3).

As the irradiation speed was 2 mm per second, the time of irradiation for each implant, depended on its dimension, with an average of 40 s in the LX1 group, for an implant of 4 mm diameter and length of 10 mm, while the irradiation took three times longer for the LX3 group. After the laser irradiation, all fixtures were returned to sterile packages, for testing.

### 2.2. Scanning Electron Microscopy

**Figure 2:**
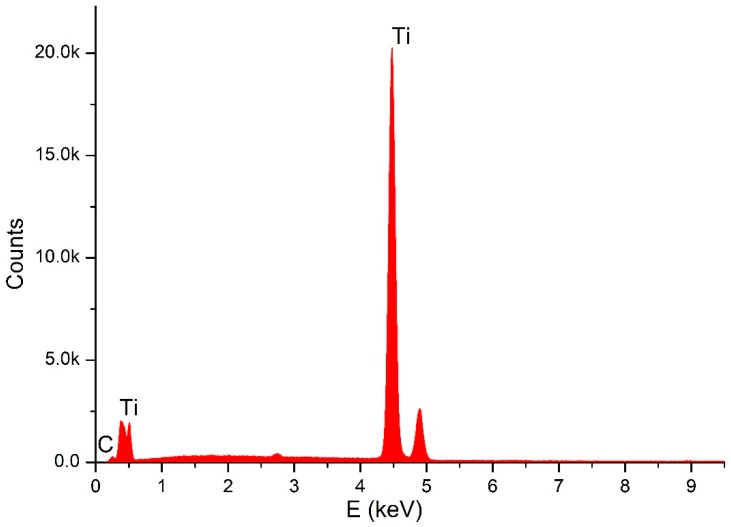
EDX for sterile implant.

**Figure 3:**
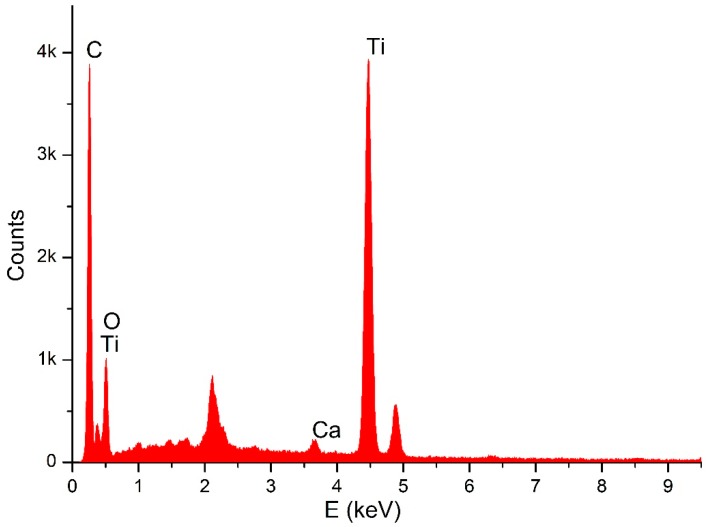
EDX for contaminated implant.

**Figure 4:**
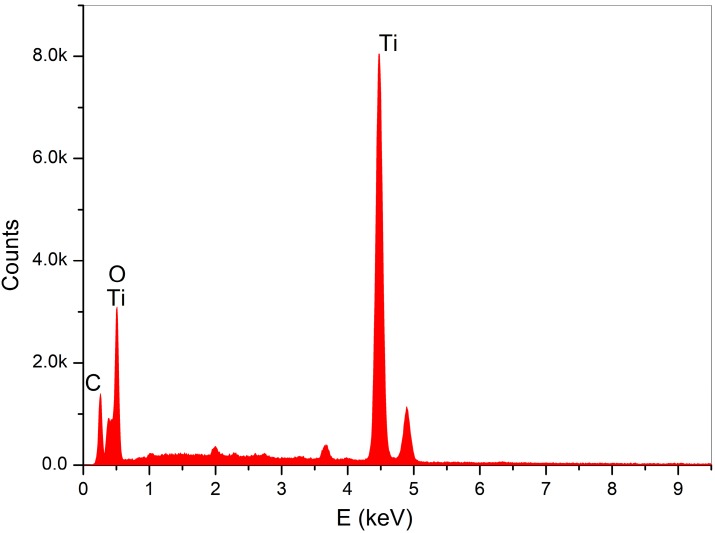
EDX for 1 passage of laser.

**Figure 5:**
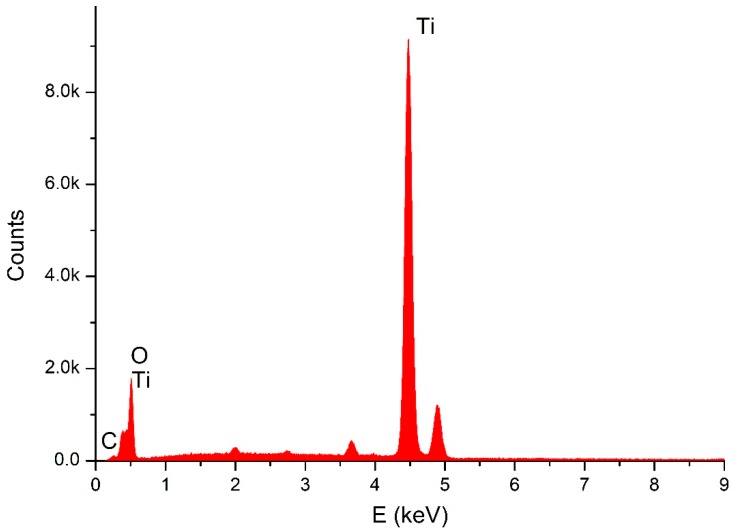
Energy-dispersive X-ray spectroscopy (EDX) for the three passages of laser.

Through SEM (Seron technologies AIS2100, Uiwang, South Korea) or EDX (EDAX Apollo detector) we analyzed the effects produced by the Er:YAG laser in one and in three passages, by evaluating the proportion of the carbon on the implant surfaces, for the sterile (control–Group A) (Figure 2), and the contaminated implant surfaces, before (Figure 3) and after the irradiation (Figure 4 and Figure 5). EDX, which is the standard technique for the local determination of the chemical composition, was used to measure the presence of carbon on the implant surfaces. The area of an EDX peak, of an element in a sample, is directly proportional to the abundance of the elements in the sample.

The implants were carefully removed from their container, using sterile tweezers, in order to prevent any contamination. Then, they were placed on a sample holder and fixed by a double-faced conductive tape on one side, while the other sides were freely facing the electron beam of the SEM. No special sample preparation was used, since the implants were already metallic and conductive, which canceled the need for any sputtering or metallization. A high-vacuum SEM was used with an acceleration voltage of 20 kV and a working distance of 25 mm, the take-off angle for the EDX was 23 degrees. The alterations on the implant surface were evaluated by inspecting the SEM images

## 3. Statistical Analysis

Statistical analyses were performed by a blind statistician, using the GraphPad Prism program (GraphPad Software, Inc., San Diego, CA, USA). Means and standard deviations (SD) of the carbon mass (%) on the implant surfaces, were reported for each group. Data showed normal and homogeneous distributions and were submitted to one-way ANOVA, followed by post-hoc Newman-Keuls test, for pairwise comparisons. A *p*-value of less than 0.05 was considered to be statistically significant.

## 4. Results

### 4.1. Analytical Results

**Figure 6:**
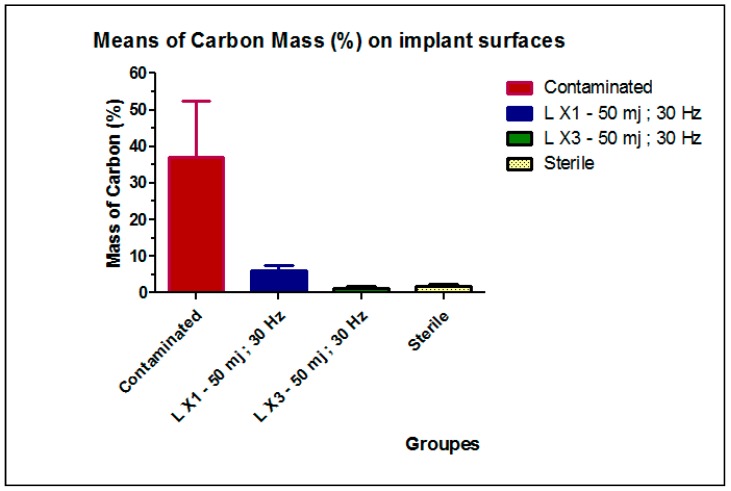
Means of the Carbon mass (%) on the implant surfaces in the sterile, contaminated, LX1, and LX3 groups.

At baseline, the contaminated Group B had a mean carbon mass (%) of 37.18 ± 15.31. This value decreased to 6.17 ± 1.45 after one laser passage and to 1.43 ± 0.41 after three laser passages (Table 2). The mean difference between the three groups (contaminated, LX1, and LX3) was statistically significant (*p* < 0.0001), as shown in Table 2. For the comparison between LX1, LX3, and the control Group A (sterile); the mean carbon mass (1.86 ± 0.68) analysis showed a significant difference between LX1 and Group A (*p* < 0.0001), while the difference between LX3 and the control group was not statistically significant (Table 3). Results are further illustrated in Figure 6. Thus, the null hypothesis was accepted, when comparing the group irradiated by three passages versus the control group of the sterile implants (*p* value > 0.05).

### 4.2. SEM Observations

**Figure 7:**
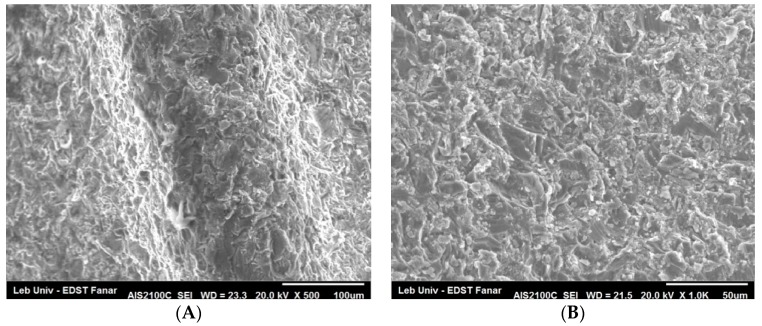

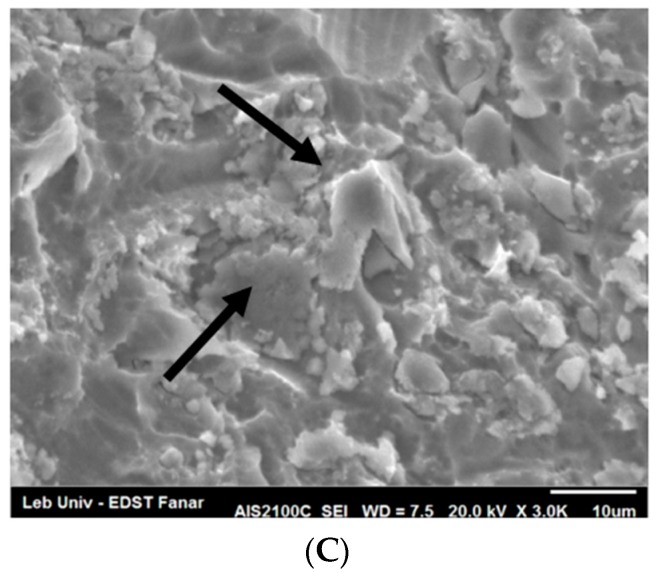
Images illustrating the characteristics of the morphology of the implant surface. Control sterile implant surface, without any conditioning, shows irregular rough surface (**A**) (×500), (**B**) (×1000), and (**C**) (×3000). High-magnification image (**C**) shows ridges and grooves on the implant surface.

**Figure 8:**
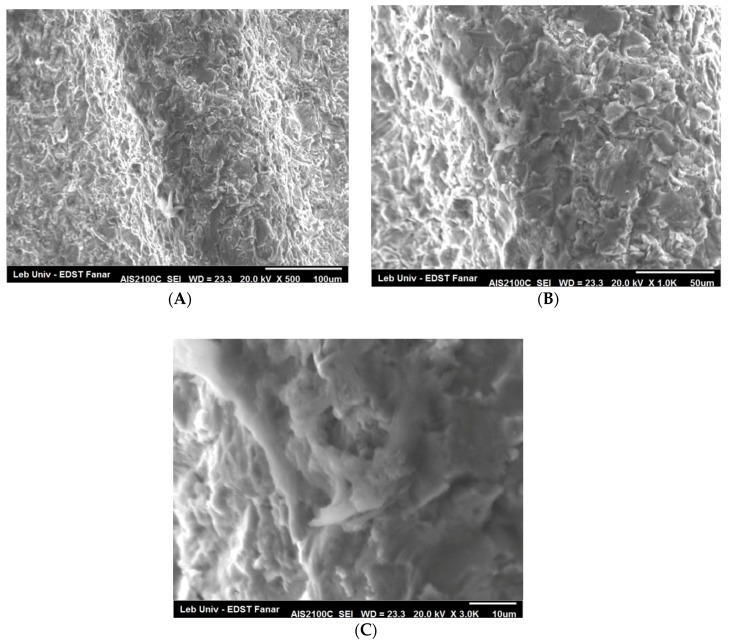
The implant surface irradiated by the Er:YAG laser, under 50 mJ/pulse, for three passages, at the magnification (**A**) (×500), (**B**) (×1000), and (**C**) (×3000).The implant surface did not get affected and the rough surface was similar to the one of the sterile implant.

The SEM examination of the implant surface after laser irradiation, in comparison with a sterile and not-treated surface, showed what has been presented in Figure 7 and Figure 8. There was no presence of any cracks or melted surface in all figures. When we used 50 mJ by three passages, the implant surface did not get affected and the rough surface was similar to the one of the sterile implant.

## 5. Discussion

Numerous procedures have been proposed for the clinical use of Er:YAG laser, in dentistry, ranging from the removal of tooth decay and cavity preparation, to many soft and hard tissue surgical procedures [35,42,43,44,45,46,47,48]. Along with these, there are other benefits, such as ablation of the target tissues and the ability to reduce bacterial contamination. Many studies have already validated the bactericidal effect of laser irradiation on the surface of the contaminated dental implants. Due to its unidirectional light and the side-firing tips, the laser beam allowed access to all threads of the implant surface, compared to the mechanical debridement by curettes which are not able to reach all parts of the surface [38].

Kreisler et al. [36] evaluated the use of Nd:YAG, Ho:YAG, Er:YAG, CO2, and GaAlAs lasers for the decontamination of implant surfaces; they concluded that Er:YAG and CO2 lasers may be used at limited powers. Several researches have studied the cleaning efficacy of the Erbium laser beam on a titanium surface, but to date there is no consensus on the appropriate parameters of lasers that are to be used during decontamination [49]. Er:YAG pulses of 300 mJ/10 Hz produce alterations to the SLA (sandblasted and acid-etched) surfaces and 500 mJ/10 Hz pulses alter the polished surfaces [36]. Galli et al. investigated the Er:YAG irradiation at two levels: 150 and 200 mJ/pulse at 10 Hz; the results of the study indicated that Er:YAG laser at these energy levels, could alter the surface profile of titanium implants and subsequently, may negatively affect the viability and the activity of the osteoblastic cells [50]. Several in vitro and in vivo studies had investigated the use of Er:YAG laser at a pulse energy of 100 mJ, with a frequency of 10 Hz, and had found it effective for the decontamination of implant surfaces [51,52]. However, it decreases the surface roughness and increases the wettability of the SLA and hydroxyapatite titanium surfaces, after one minute of irradiation [44]. Nevertheless, another study concluded that no surface alteration was detected after irradiation at an intensity of 100 mJ/pulse, at 10 Hz, for 1 min, while the titanium’s roughness was affected after 1.5 min of Er:YAG irradiation [41]. This result was already concluded by Kim et al., who recommended the application settings of 100 mJ/pulse, at 10 Hz and for less than two minutes, to detoxify the implant surface without causing any surface modifications [53]. The energy level of 50 mJ has shown efficiency in removing plaque and calculus on the implant abutments, without injuring their surfaces [38]. When the pulse energy and irradiation time increased, greater surface alterations, including surface flattening and microfractures were observed [41]. In the present study we used the same low-level energy of Er:YAG (50 mJ) on a rough implant surface, to test it by a single and multiple passages. The results were promising as Group LX3 (3 passages) was almost perfectly cleaned, in comparison to the negative control group, in which no contamination technique was used. The value of carbon mass decreased after one laser passage but analysis showed a significant difference between LX1 and the control group (*p* < 0.05), while the difference between LX3 and the control group was not statistically significant (Table 3). Therefore, irradiation by one passage, with the mentioned parameters (50 mJ), did not show efficiency in the implant surface decontamination. A great difference was noted between LX1 (one passage) and LX3. Reduction in the presence of carbon in LX3, in comparison to LX1, was probably caused by the multiple passages of laser beam. Thus, multiple irradiations with Er:YAG laser produced the removal of all particles.

The control of the energy density is a key factor of success for any treatment by laser irradiation [54]. Taniguchi et al. used a pulse energy of 30–50 mJ/pulse at a repetitive rate of 30 Hz. They had concluded that Er laser irradiation at pulse energies below 30 mJ/pulse and 30 Hz, is effective for debriding microstructure surfaces and the fluency being 10.6 J/cm^2^ [55], which was greater than the value used in our study. These findings suggest that in our experiment, the reduction of carbon presence on the implant surface was rather due to the multiple passages of laser.

No surface change was seen in the irradiated groups (Figure 6); this result was in agreement with a previous study that revealed no distinct morphological alterations under 50 mJ. Due to a serious concern regarding implant overheating, the laser beam was used with concomitant water spray irrigation under air 6 and water 4, to minimize thermal damage, by keeping the irradiated area moist [56]. We cannot completely rule out the effect of water irrigation on removing some contaminants, but it is impossible to get a surface as clean as a sterile implant’s surface. Although, Park et al. concluded in their study that the use of a dental water jet didn’t show any efficacy in the dental implant decontamination [57]. The impact of the laser tip on heat generation, during implant decontamination, has received little attention. The tip used in our study was a sapphire tip. Romanos et al. have studied the influence of two laser tips (sapphire chisel and radial firing perio) on temperature change after laser irradiation; they concluded that sapphire may be preferable for implant debridement [8].

In addition, one of the key factors that regulate the regime and efficacy of laser decontamination is the laser pulse duration. The VSP (very short pulse) Er:YAG laser can be operated at adjustable pulse duration, from super short pulses (SSP) that are ideal for the precise ablation of hard tissue, to very long pulses (VLP) for more coagulative soft tissue procedures. This technological solution provides nearly square-shaped power pulses, the duration of which can be conveniently controlled over a wide range of pulse durations [58]. In our experiment, the beneficial effect of the Er:YAG laser in implant decontamination was particularly pronounced when the laser was set to operate at super-short pulses (SSP, 50 µs) and a low-pulse energy, which provided an extremely precise laser ablation with minimal thermal effect, since there was a very short duration to escape the ablated volume [59]. 

Folwaczny et al. concluded that the efficiency was maximal at an angle of 90 degrees, during the removal of a root substance [60]. In this study, for maximum energy application, the irradiation angle was 90 degrees at a focal distance of 2 mm, however, the implant design was not a smooth titanium plate but a dental implant thread surface, and the laser illumination angle limitation caused by the threads could make the clinical application different from in vitro experiments. Our study has shown that the decontamination of implants is possible with Er:YAG, under certain conditions, particularly, the condition of angulation of 90 degrees. Having a 90° angle may not always be respected, in situ, therefore, there is probably a risk of reduced effectiveness in cleaning the implant. Accordingly, it is interesting to use the adapted Er:YAG tips allowing the delivery of a beam at 90° into the pitch of the threads, for effective decontamination.

The previous studies focused on the activity of the Er:YAG laser, against planktonic bacteria [15,35,61]. Our in-vitro study has evaluated the efficiency of Er:YAG laser to remove carbon and to get a surface composition comparable to the uncontaminated implant surface with decreased amounts of carbon.

The percentage mass of carbon present on the implant’s surface is a measure of its contamination. The lower the percentage, the cleaner was the implant. The results of our study showed similarity in the count of carbon between the sterile and laser-irradiated implants (by three passages), in comparison with contaminated ones and one passage lasing. Er:YAG was efficient to clean the implant surfaces without altering the titanium surface, by an irradiation of 50 mJ/pulse at 30 Hz, for three passages. In addition, this procedure was very fast since an implant may be cleaned in about one minute, depending on the exposed area.

Further studies should be directed to evaluate the biocompatibility and the cellular adhesion of the titanium surfaces, after their cleaning using similar Er:YAG irradiation conditions.

## 6. Conclusions

Our results suggest that the Er:YAG laser could be considered an effective tool for the decontamination of implant surfaces when used with an energy of 50 mJ, frequency of 30 Hz, in the super short pulse mode (SSP), and a fluency of 3.76 J/cm^2^. When the irradiation speed was 2 mm/s, the laser irradiation by three passages was very efficient.

## Figures and Tables

**Table 1:** Distribution of the experimental and the control groups.

Groups	Surface
A (sterile) (n = 30)	Control: No contamination/No irradiation
B (n = 60)	Contaminated implants
LX1 (n = 30)	Decontamination by irradiation: one passage
LX3 (n = 30)	Decontamination by irradiation: multiple passages

**Table 2:** Mean values (SD) of the Carbon mass (%) analysis according to the groups.

Groups	Contaminated	L X1 50 mJ; 30 Hz	L X3 50 mJ; 30 Hz	Sterile
Number of values	230	235	235	235
Mean (SD)	37.18 (15.31) ^a^	6.17 (1.45) ^b^	1.43 (0.41) ^c^	1.86 (0.68) ^c^
95% CI	34.24–40.11	5.06–7.28	0.92–1.93	1.64–2.08

Lowercase superscript letters indicate statistically significant differences (Newman-Keuls; *p* < 0.05) between groups. SD: Standard deviation; CI: Confidence interval.

**Table 3:** Table showing the mean differences and *p*-values of each of the two tested-group. The difference is significant between all types of groups, except between LX3 and the sterile group. *** means the difference is highly significant (0.1 %).

*p* Value Summary	***			
Do the variances differ signif. (*p* < 0.05)	Yes			
ANOVA Table	SS	Df	MS	
Treatment (between columns)	65,790	3	21,930	
Residual (within columns)	3129	116	26.97	
Total	68,920	119		
Newman-Keuls Multiple Comparison Test	Mean Diff.	Q	Significant? *p* < 0.05?	Summary
L X3-50 mj; 30 Hz vs. Contaminated	−55.82	58.87	Yes	***
L X3-50 mj; 30 Hz vs. L X1-50 mj; 30 Hz	−5.213	5.498	Yes	***
L X3-50 mj; 30 Hz vs. Sterile	−0.6283	0.6627	No	ns
Sterile vs. Contaminated	−55.19	58.21	Yes	***
L X1-50 mj; 30 Hz vs. Sterile	−4.585	4.836	Yes	***
L X1-50 mj; 30 Hz vs. Contaminated	−50.61	53.37	Yes	***
